# Scoliosis Causing Cervical Dystonia in a Chiropractic Office

**DOI:** 10.7759/cureus.35802

**Published:** 2023-03-05

**Authors:** Eric Chun-Pu Chu, Wai Ting Lee, Damien Ming Yan Tam, Natalie Y Ng, Aimen B Nur

**Affiliations:** 1 New York Medical Group (NYMG) Chiropractic Department, EC Healthcare, Hong Kong, HKG; 2 Chiropractic Department, EC Healthcare, Kowloon, HKG; 3 Chiropractic Department, EC Healthcare, Yuen Long, HKG; 4 Chiropractic Department, EC Healthcare, Mong Kok, HKG

**Keywords:** idiopathic scoliosis, primary dystonia, focal dystonia, adolescent idiopathic scoliosis (ais), cervical dysonia, chiropractic therapy

## Abstract

Cervical dystonia is a movement disorder characterized by continuous and involuntary muscular contractions that result in aberrant head and neck motions or postures. A recent study indicates that persons with a history of scoliosis may be at a higher risk of acquiring cervical dystonia later in life. Although muscular tension and contraction abnormalities are linked in both illnesses, the pathophysiological pathways linking these two ailments are not entirely understood. A 13-year-old boy previously diagnosed with adolescent idiopathic scoliosis developed symptoms of cervical dystonia, including moderate neck pain, left-sided migraines, and tingling in the neck and shoulders. During the course of three months, the patient attended 16 chiropractic therapy sessions. He reported slow but considerable improvements in his symptoms, such as the recovery of normal cervical range of motion, decreases in neck discomfort and accompanying headaches as well as paresthesia, and enhancements in sleep quality, daily functioning, and learning capacities. The patient's clinical and radiographic improvements show that chiropractic spinal manipulation may assist in reducing pain and improving spine alignment and mobility in these circumstances. To further investigate the efficacy and safety of chiropractic therapy for the treatment of cervical dystonia, particularly in the setting of associated scoliosis, more study with bigger patient populations is required.

## Introduction

Cervical dystonia, also known as spasmodic torticollis, is a neurological disease characterized by aberrant head postures and movements caused by involuntary muscle spasms in the neck. It is a special form of focal dystonia that affects the neck. The direct cause of cervical dystonia is unclear, while it is considered to entail a combination of genetic vulnerability and environmental variables. The dystonic muscular contractions and spasms lead to twisting and aberrant posture of the head and neck [1]. However, secondary cervical dystonia may appear as a consequence of concomitant neurological disease, any other disorder, or scoliosis [2,3].

Scoliosis is more likely to appear in patients with cervical dystonia in late childhood or early adolescence [4]. Contraction of antagonistic muscles is the general pathophysiological feature of dystonia [2]. According to the most recent research, scoliosis of the spine causes the muscles surrounding the spine to become more tense globally [5]. Individuals with a history of scoliosis are more likely to experience cervical dystonia [3]. After clinical management of scoliosis, researchers found a decreased correlation between a family history of dystonia and cervical dystonia, suggesting a potential connection between scoliosis and dystonia [3].

The insufficient study has been conducted on the use of chiropractic therapy in the treatment of cervical dystonia; therefore, additional research is required to determine the full potential of this approach. Chiropractic treatment can effectively treat neuromusculoskeletal disorders with minimal adverse effects [6], and prior case studies have suggested that chiropractic therapy may be an effective alternative or complementary treatment option for individuals with cervical dystonia [7-9]. The patient's improvement in spinal alignment and reduction of muscular tension in the neck and surrounding areas, as well as relief in chronic neck pain, headaches, and radicular pain, suggested that chiropractic therapy may be effective in treating these disorders.

This case report describes a patient with adolescent idiopathic scoliosis who developed symptoms of cervical dystonia, which was diagnosed and treated by chiropractic therapy. The improvement in scoliosis, relief of acute neck pain, and increase in overall quality of life suggest that chiropractic therapy may be an effective complementary therapy option for individuals with adolescent idiopathic scoliosis who developed symptoms of cervical dystonia.

## Case presentation

A 13-year-old boy with scoliosis presented with the primary complaints of moderate neck pain and an awkward chin position lasting for two weeks. He reported that the symptoms had begun after he sneezed and heard a popping sound on his neck while eating breakfast. He described the associated discomfort as a persistent ache, radiating from the sides of the neck down to the occiput and shoulders. The pain gradually worsened over the day and reached a maximum level of 4 out of 10 on a numeric pain scale by the evening. Other symptoms included left-sided headaches, numbness, weakness, and tingling in the neck. The pain was exacerbated by the prolonged use of mobile phones with the head bowed down. He was unable to turn his head fully to the left, and his neck movement and posture worsened. He denied difficulty breathing, changes in bladder and bowel habits, weakness, and renal or cardiac problems. He further denied sleeping in an awkward neck position the night before the symptoms. His quality of life and sleep were significantly affected by the symptoms, and learning difficulties were triggered by restriction and constriction of the neck. He was diagnosed with mild adolescent idiopathic scoliosis one year prior. His family physician initially treated his neck pain with a muscle relaxant and nonsteroidal anti-inflammatory drugs (NSAIDs) one day after the incident. However, the discomfort was exacerbated over the course of a week, prompting the patient to attend the emergency room. Cervical radiography revealed a scoliotic curve in the thoracic and lumbar spine, and the patient was discharged with a diagnosis of cervical dystonia. Owing to his worsening symptoms, which did not respond to treatment, he sought chiropractic therapy.

**Figure 1:**
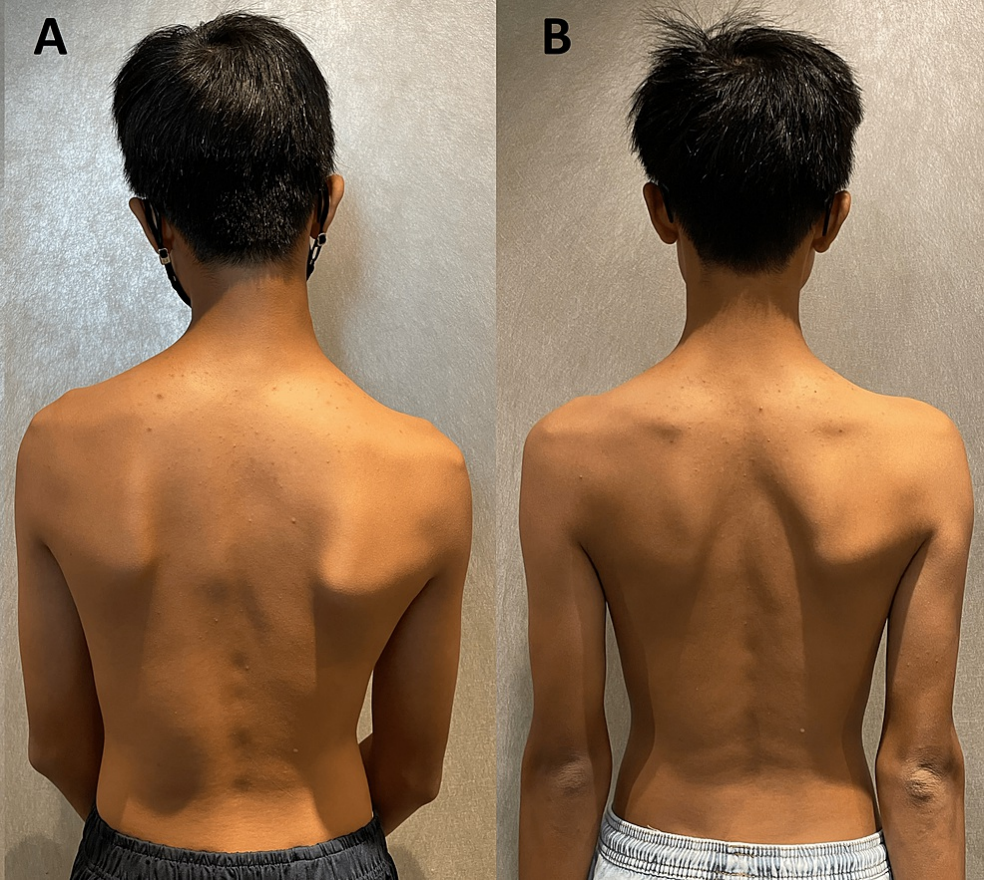
Photo of the back (A) Posture analysis identified uneven shoulders, scapulae, and iliac crest, with the head not centered over the pelvis before treatment. (B) Improvement and balance posture were identified after the treatment.

**Figure 2:**
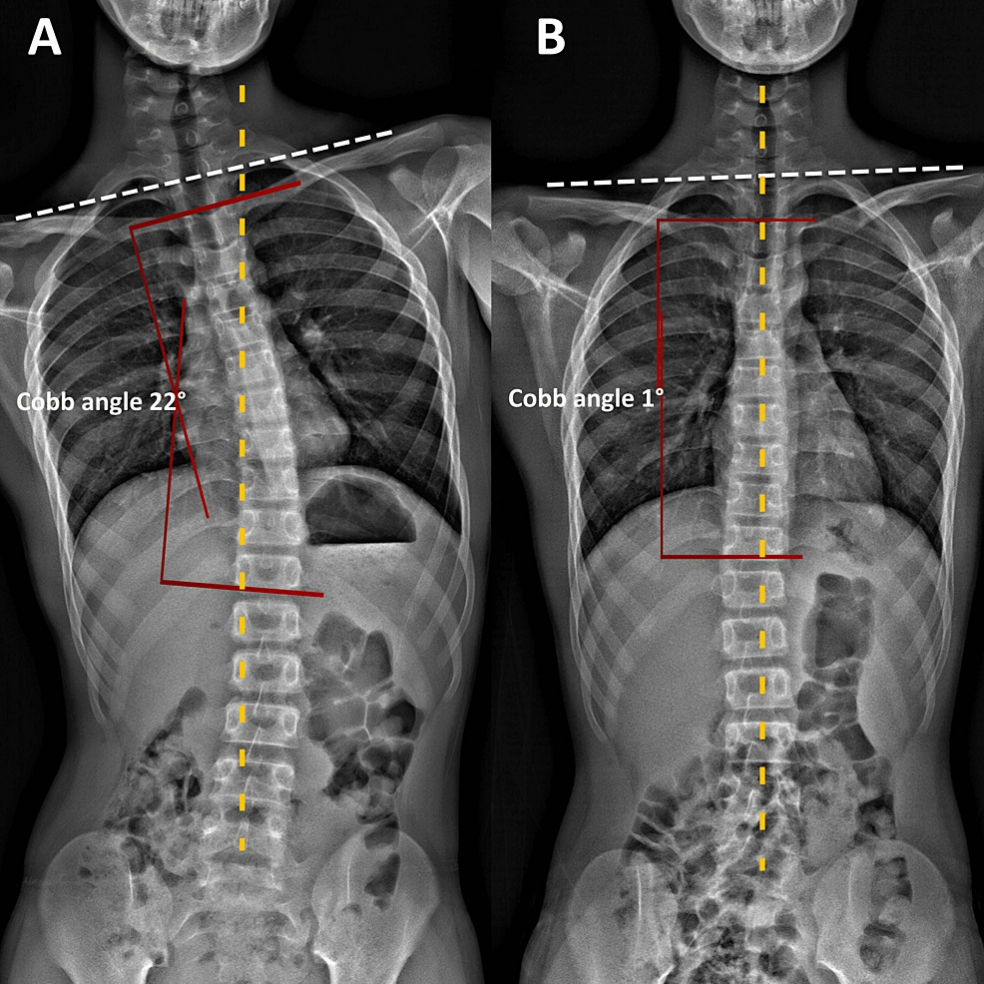
Full-spine radiographs (frontal view) (A) Pre-treatment radiographs revealed an uneven spine, clavicles (dash lines), and pelvic and trunk shift. A scoliotic curve was measured at Cobb angle 22° in the absence of structural osseous anomalies before treatment. (B) Post-treatment radiographs revealed improvement in spinal alignments, and clavicles (dash lines) balance. Cobb angle reduced from 22° to 1°.

**Figure 3:**
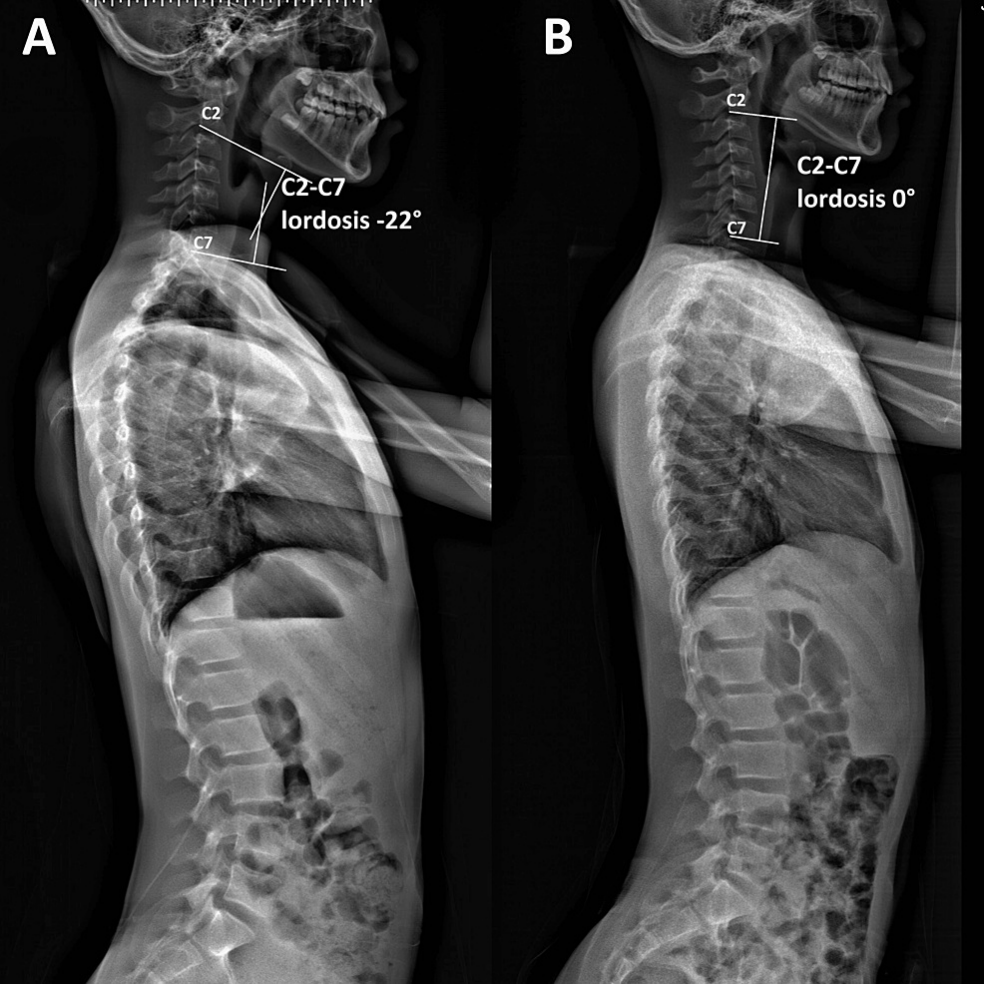
Full-spine radiographs (sagittal view) (A) Pre-treatment radiographs revealed reversed cervical lordosis. Using the Cobb angle (white lines), the C2-C7 spine’s global curvature was initially measure as –22°, indicating a reversed cervical lordosis. (B) At a 3-month follow-up, the cervical lordosis was corrected by 22° (0° vs. –22°) using the C2-C7 spine Cobb angle.

The patient is 168 cm and 56 kg. He presented with a slouched posture and a regular gait. Observation from the back showed that his shoulders, scapulae, and iliac crest were observed to be asymmetrical, and the head was not centered over the pelvis (Figure 1A). Orthopedic testing revealed a positive result on the Adams forward bend test for scoliosis and a positive result on Spurling's maneuver for neck pain without radiculopathy. Upon initial examination, his cervical range of motion was limited to extension, flexion, and left rotation. His left sternocleidomastoid, upper trapezius, and bilateral suboccipital and paraspinal muscles exhibited spasms and pain on palpation. Intersegmental joint restrictions were indicated in segments C5-T2. Normal leg length measurements were taken for his lower extremities, and his feet showed no evidence of a high arch. Laboratory results revealed that electrolytes, blood count, and creatinine levels were normal. Full-spine radiographs revealed uneven spine, scapula, pelvis, and trunk shifts. Reversed cervical lordosis and a scoliotic curve were measured at Cobb 22° in the absence of structural osseous anomalies (Figures 2A, 3A). The chiropractor’s differential diagnoses included spinal stenosis, Parkinson's Disease, Wilson disease, adult-onset idiopathic torticollis, and post-traumatic dystonia. Considering the patient’s history and clinical presentation, which had worsened over the past two weeks despite conservative care, the chiropractor recommended cervical magnetic resonance imaging (MRI) to rule out spinal stenosis. The patient’s MRI returned the next day and was unremarkable.

Spinal manipulative therapy, thermal ultrasonography therapy, mechanical vibration massage (G5 ® Massage Machine, General Physiotherapy, Inc., USA), and motorized flexion-distraction therapy were applied as chiropractic treatment. To restore muscle function, joint motion, and intersegmental dysfunction, the initial phase of treatment consisted of three sessions per week for three weeks. In addition, the patient was instructed in ergonomics regarding workstation design, repetitive tasks, and mobile device usage limitations. In the first week, the patient's neck pain decreased to a moderate level of severity. At the conclusion of the first phase of treatment, he also reported recovery of his cervical motion, as well as improvements in sleep quality and learning disabilities. The frequency of therapy sessions was decreased to once per week for an additional two months. Three months after the initial consultation, the patient reported no residual symptoms or complete neck movement. No pathogenic or treatment-related adverse effects were observed, and the patient’s abnormal posture had returned to normal (Figure 1B). Thoracolumbar scoliosis and cervical lordosis are significantly improved (Figures 2B, 3B).

## Discussion

Studies in the literature have suggested a strong correlation between cervical dystonia and scoliosis. For example, a genetic link between scoliosis and cervical dystonia has been suggested due to a higher prevalence of adolescent-onset scoliosis among patients with primary adult-onset cervical dystonia compared to the general population [10]. Patients with congenital cervical dystonia often develop secondary cervicothoracic scoliosis [11,12], which may result in secondary scoliosis in the long term. The reasons for this correlation are not fully understood, but it may be that the same genetic or developmental factors that lead to a higher risk of scoliosis also increase the risk of cervical dystonia. Alternatively, the altered biomechanics and posture associated with scoliosis could place extra stress on the neck and contribute to the development of cervical dystonia in susceptible individuals.

As the history is related to trauma, information on the prevalence of patients with cervical dystonia related to trauma is 5%-21% [13]., and the severity of the scoliosis curve frequently rises quickly in teenage dystonia patients [14]. The effectiveness of physiotherapy as a treatment for cervical dystonia remains to be established [15]. Patients with curves greater than 15° who are skeletally immature are at a high risk of developing muscular dystonia [16,17]. Our patient in the current study had scoliosis at the age of 13 and had a curvature of more than 20 degrees upon presentation, indicating a significant risk of scoliosis progressing rapidly. According to long-term follow-up studies, congenital muscular torticollis has been linked to secondary scoliosis, [10]. Therefore, in order to prevent scoliotic deformities and disabilities, it is essential to diagnose dystonia as soon as possible in adolescents [18,19].

Treatment choice depends on disease severity, specific symptoms, and patient preferences. Treatment options for cervical dystonia include medications, botulinum toxin injections, and surgery. Oral medications such as muscle relaxants, benzodiazepines, and anticholinergics can help reduce muscle spasms and pain [20,21]. Botulinum toxin injections directly on neck muscles can provide targeted relief from muscle contractions, but they also have adverse effects, including dry mouth and dysphagia, and require repeated injections [22]. There is also limited evidence from randomized clinical trials regarding the safety and efficacy of repeated injections [23]. The effectiveness of physiotherapy as a treatment for cervical dystonia remains unknown [14]. As a last resort, surgical sternocleidomastoid release for congenital cervical dystonia is effective for scoliosis before the patient reaches the end of the growth period [11].

The present case study demonstrates the effectiveness of chiropractic therapy in alleviating neck pain and reducing scoliotic curves in patients with cervical dystonia. The patient in this study responded positively to the three months of care provided, reporting the recovery of his cervical motion, improvements in sleep quality, learning disabilities, and quality of life. The frequency of therapy sessions was later decreased to once a week for two additional months, after which the patient reported no residual symptoms and complete neck movement. No adverse effects were observed, and the patient's abnormal posture returned to normal with significant improvement in cervical lordosis and thoracolumbar scoliosis. The efficacy of chiropractic therapy to correct scoliosis, especially over the long term [24], is partially demonstrated by the literature [25,26], which showed that three months of treatment resulted in radiographic benefits, improvements in numeric pain score, and reduction of medication, particularly for skeletally immature patients. Scoliosis should be regarded as a significant cause of cervical dystonia.

## Conclusions

This case study highlights the possible advantages of chiropractic care in alleviating neck pain and reducing scoliotic curves in patients with cervical dystonia. Chiropractic care may be a useful alternative or supplemental treatment for people with cervical dystonia, as evidenced by the patient's improvement in cervical pseudo-scoliosis and reduction of acute neck pain. To prove the effectiveness and safety of chiropractic therapy and to ascertain its full potential for the treatment of this ailment, additional research with bigger patient populations is required.

## References

[1] Harrison DE, Harrison DD, Colloca CJ, Betz J, Janik TJ, Holland B (2003). Repeatability over time of posture, radiograph positioning, and radiograph line drawing: an analysis of six control groups. J Manipulative Physiol Ther.

[2] Morningstar MW, Joy T (2006). Scoliosis treatment using spinal manipulation and the Pettibon Weighting System: a summary of 3 atypical presentations. Chiropr Osteopat.

[3] Defazio G, Abbruzzese G, Girlanda P (2003). Primary cervical dystonia and scoliosis: a multicenter case-control study. Neurology.

[4] Doménech J, Tormos JM, Barrios C, Pascual-Leone A (2010). Motor cortical hyperexcitability in idiopathic scoliosis: could focal dystonia be a subclinical etiological factor?. Eur Spine J.

[5] Albanese A, Bhatia K, Bressman SB (2013). Phenomenology and classification of dystonia: a consensus update. Mov Disord.

[6] Chu EC, Trager RJ, Lee LY, Niazi IK (2023). A retrospective analysis of the incidence of severe adverse events among recipients of chiropractic spinal manipulative therapy. Sci Rep.

[7] Chu EC, Chen AT, Chiang R (2022). Chiropractic care of Parkinson's disease and deformity. J Med Life.

[8] Brurberg KG, Dahm KT, Kirkehei I (2019). Manipulation techniques for infant torticollis. Tidsskr Nor Laegeforen.

[9] Chu EC, Lo FS, Bhaumik A (2020). Secondary atlantoaxial subluxation in isolated cervical dystonia-a case report. AME Case Rep.

[10] O'Riordan S, Lynch T, Hutchinson M (2004). Familial adolescent-onset scoliosis and later segmental dystonia in an Irish family. J Neurol.

[11] Kim JH, Yum TH, Shim JS (2019). Secondary cervicothoracic scoliosis in congenital muscular torticollis. Clin Orthop Surg.

[12] Park JI, Kee JH, Choi JY, Yang SS (2021). Is longstanding congenital muscular torticollis provoking pelvic malalignment syndrome?. Children (Basel).

[13] Samii A, Pal PK, Schulzer M, Mak E, Tsui JK (2000). Post-traumatic cervical dystonia: a distinct entity?. Can J Neurol Sci.

[14] Kukurin GW (2004). Reduction of cervical dystonia after an extended course of chiropractic manipulation: a case report. J Manipulative Physiol Ther.

[15] De Pauw J, Van der Velden K, Meirte J (2014). The effectiveness of physiotherapy for cervical dystonia: a systematic literature review. J Neurol.

[16] Ciftdemir M, Copuroğlu C, Ozcan M, Ulusam AO, Yalnız E (2012). Non-operative treatment in children and adolescents with atlantoaxial rotatory subluxation. Balkan Med J.

[17] Pagé I, Descarreaux M (2019). Effects of spinal manipulative therapy biomechanical parameters on clinical and biomechanical outcomes of participants with chronic thoracic pain: a randomized controlled experimental trial. BMC Musculoskelet Disord.

[18] Bočková M, Chládek J, Šímová L, Jurák P, Halámek J, Rektor I (2013). Oscillatory changes in cognitive networks activated during a three-stimulus visual paradigm: an intracerebral study. Clin Neurophysiol.

[19] Pu Chu EC, Kai Huang KH (2017). Bridging the gap between observation and brace treatment for adolescent idiopathic scoliosis. J Family Med Prim Care.

[20] Strong RE, Marchant BK, Reimherr FW, Williams E, Soni P, Mestas R (2009). Narrow-band blue-light treatment of seasonal affective disorder in adults and the influence of additional nonseasonal symptoms. Depress Anxiety.

[21] Ghanem I, El Hage S, Rachkidi R, Kharrat K, Dagher F, Kreichati G (2008). Pediatric cervical spine instability. J Child Orthop.

[22] Novaretti N, Cunha AL, Bezerra TC (2019). The prevalence and correlation of non-motor symptoms in adult patients with idiopathic focal or segmental dystonia. Tremor Other Hyperkinet Mov (N Y).

[23] Marques RE, Duarte GS, Rodrigues FB (2016). Botulinum toxin type B for cervical dystonia. Cochrane Database Syst Rev.

[24] Brashear A, Lew MF, Dykstra DD (1999). Safety and efficacy of NeuroBloc (Botulinum Toxin Type B) in type A-responsive cervical dystonia. Neurology.

[25] Pu Chu EC, Chakkaravarthy DM, Huang KH, Ho VW, Lo FS, Bhaumik A (2020). Changes in radiographic parameters following chiropractic treatment in 10 patients with adolescent idiopathic scoliosis: a retrospective chart review. Clin Pract.

[26] Oakley PA, Kallan SZ, Harrison DE (2022). The reduction of high thoracic scoliosis in adults by mirror image(®) blocking: a Chiropractic BioPhysics(®) case series. J Phys Ther Sci.

